# The Use of San-Huang-Xie-Xin-Tang Reduces the Mortality Rate among Breast Cancer Patients

**DOI:** 10.3390/cancers15041213

**Published:** 2023-02-14

**Authors:** Daniel Winardi, Chieh-Hsin Wu, Jen-Huai Chiang, Yung-Hsiang Chen, Ching-Liang Hsieh, Juan-Cheng Yang, Yang-Chang Wu

**Affiliations:** 1 Graduate Institute of Integrated Medicine, College of Chinese Medicine, China Medical University, Taichung 40402, Taiwan; 2 Division of Neurosurgery, Department of Surgery, Kaohsiung Medical University Hospital, Kaohsiung 807, Taiwan; 3 Department of Surgery, School of Medicine, College of Medicine, Kaohsiung Medical University, Kaohsiung 807, Taiwan; 4 Management Office for Health Data, Clinical Trial Research Center, China Medical University Hospital, Taichung 404, Taiwan; 5 Department of Psychology, College of Medical and Health Science, Asia University, Taichung 413, Taiwan; 6 Department of Chinese Medicine, China Medical University Hospital, Taichung 404, Taiwan; 7 Graduate Institute of Integrated Medicine, Graduate Institute of Acupuncture Science, College of Chinese Medicine, China Medical University, Taichung 404, Taiwan; 8 Chinese Medicine Research and Development Center, China Medical University Hospital, Taichung 404, Taiwan; 9 School of Chinese Medicine, College of Chinese Medicine, China Medical University, Taichung 40604, Taiwan; 10 Department of Medical Laboratory Science and Biotechnology, College of Medical and Health Science, Asia University, Taichung 413, Taiwan

**Keywords:** breast cancer, mortality, population, risk, San-Huang-Xie-Xin-Tang (SHXXT)

## Abstract

**Simple Summary:**

Since San-Huang-Xie-Xin-Tang (SHXXT) is a potent anti-tumor therapy and breast cancer is the most common cause of cancer deaths worldwide, in the present retrospective cohort study, we explore the influence of SHXXT and its constituents on the mortality rate by analyzing 5387 breast cancer patients taking SHXXT and its constituents and 5387 breast cancer patients not using SHXXT and its constituents. Our study confirms SHXXT and its constituents are a useful alternative therapy to decrease the breast cancer mortality rate. In particular, the use of SHXXT is more effective than the use of only one constituent. With the increasing cumulative days of use and the annual average dose, the anti-tumor effect is more pronounced.

**Abstract:**

Globally, breast cancer is the most common cause of cancer deaths. In Taiwan, it is the most prevalent cancer among females. Since San-Huang-Xie-Xin-Tang (SHXXT) exerts not only an anti-inflammatory but an immunomodulatory effect, it may act as a potent anti-tumor agent. Herein, the study aimed to explore the influence of SHXXT and its constituents on the mortality rate among breast cancer patients in Taiwan regarding the component effect and the dose–relationship effect. By using the Taiwan National Health Insurance (NHI) Research Database (NHIRD), the study analyzed 5387 breast cancer patients taking Chinese herbal medicine (CHM) and 5387 breast cancer patients not using CHM. CHM means SHXXT and its constituents in the study. The Kaplan–Meier method was utilized to determine the mortality probabilities among patients. Whether the CHM influences the mortality rate among patients was estimated by Cox proportional hazard regression analysis. The use of CHM could lower the cancer mortality rate by 59% in breast cancer patients. The protective effect was parallel to the cumulative days of CHM use and the annual average CHM dose. In addition, the mortality rate was lower in patients who used SHXXT compared to those who only used one of its constituents. SHXXT and its constituents were all promising therapeutic weapons against breast cancer.

## 1. Introduction

Around the world, cancer is the top reason of death. During the past two decades, the annual incidence of breast cancer has doubled or tripled in Asia [1]. In Taiwan, breast cancer is also the most common cancer among females with increasing prevalence and incidence [2,3,4]. The etiology of breast cancer remains unsettled, which is attributable to genetic, hormonal, or reproductive factors [5]. Although the standard treatments for breast cancer patients include radiotherapy, chemotherapy, and target therapy [6], many patients still seek for help with complementary and alternative medicine. In Taiwan, adult cancer patients who used traditional Chinese medicine (TCM) had a lower cancer mortality rate than those without using TCM (adjusted hazard ratio (HR) = 0.69). Chinese herbal medicine (CHM) and acupuncture are the most common alternative choices [7,8,9,10]. TCM elicits a protective effect against breast cancer; accordingly, consumption of Chinese herbs can reduce the incidence of invasive breast cancer [11]. In Taiwan, a large proportion of patients receive CHM and Western medicine concurrently against breast cancer [12]. 

Since the inflammatory signals can trigger the formation of a tumor microenvironment, several cancers arise from chronic inflammation [13]. Being a CHM, San-Huang-Xie-Xin-Tang (SHXXT) is composed of three herbal medicines, *Rhizoma Rhei*, *Radix Scutellaria*, and *Rhizoma Coptidis* at a ratio of 2:1:1 or 1:1:1 [14]. It serves as a possible therapeutic choice for hepatitis C virus (HCV) infection [15], hypertension [16,17,18], septic shock [19], neuronal damage [20], gastrointestinal (GI) disorders [21,22], acute lung injury [23], and cardiomyocyte injury [24]. SHXXT can decrease COX2 and NF-κB induction to suppress the replication of HCV [15]. Through the expression of COX2, ROCK-II, and PDE5, SHXXT ameliorated U46619-induced systemic and pulmonary arterial blood hypertension [18]. Mediating by the formation of iNOs, COX2, and PGE2, SHXXT can prevent hypotension in LPS-treated rats [19]. In the neuroblastoma SH-SY5Y cells, SHXXT can prevent the formation of ROS and inflammatory response against neurotoxicity [20]. In experimentally induced GI motility dysfunction mice, SHXXT is a novel prokinetic agent to alleviate GI motility dysfunction in a dose-dependent manner [21]. In *Helicobacter pylori* infection, SHXXT can induce the anti-inflammatory effect through inhibiting the activation of NF-κB, the production of iNOS, COX-2, and IL-8 in human gastric epithelial AGS cells [22]. In addition, SHXXT attenuates inflammatory responses by decreasing the expression of IL-1β, iNOS, and TNF-α in lipopolysaccharide-induced rat lung injury [23]. It can protect rats’ cardiomyocyte against apoptosis through eNOs and MAPK pathways after ischemia-reperfusion injury [24]. Besides, series studies have demonstrated that SHXXT has immunomodulatory, neuroprotective, and a potent anti-cancer effect [25,26,27,28]. Since, up till now, no studies have elucidated the relevance of SHXXT for breast cancers in detail, this population-based study aimed to investigate the effect of SHXXT and its constituents on breast cancer patients by analyzing the Taiwan National Health Insurance Research Database (NHIRD). Beside the impact of single constituent or compounds on cancer mortality rate, the study also clarified the dose–relationship effect based on the stratification of cumulative days of CHM use and the annual average CHM dose.

## 2. Materials and Methods

### 2.1. Data Source

From March 1995 till now, the Taiwan national health insurance (NHI) program has covered 99.6% of residents. All medical records reimbursed by the NHI were included in the NHIRD, in which the registry of beneficiaries, ambulatory and inpatient care claims data, and the registry of catastrophic illness were the source of the database. In the study, study subjects were recruited from the ambulatory and inpatient claims data liked with the registry of Catastrophic Illness during 2000 to 2010 and followed to the end of 2011.

### 2.2. Study Design and Cohort

This study aimed to evaluate the association between breast cancer mortality rate and the use of CHM, including SHXXT or one of its constituents (*Rhizoma Coptidis*, *Rhizoma Rhei*, or *Radix Scutellaria*). From the Registry for Catastrophic Illness database, 79,510 female patients who were newly diagnosed with breast cancer (International Classification of Disease, Ninth Revision, Clinical Modification (ICD-9-CM) = 174) during 2000–2010 were enrolled. The ICD-9-CM codes were identified by Chinese medicine physicians. The index date was the date of new diagnosis with breast cancer. They were followed up until the end of December 2011, death, or withdrawn from the insurance within a year of the follow-up period. Subjects younger than 18 years old or incomplete data were excluded from the analysis (n = 273). Subjects who withdraw from the insurance within a year of the follow-up period were also excluded (n = 51). A patient who took SHXXT or one of the constituents for more than 30 days was set as a CHM user (n = 5505). A patient without using CHM, acupuncture, or manipulative records was identified as a non-CHM user (n = 10,153). Subsequently, in order to match the two populations equally, the CHM user and the non-CHM user identified 5387 breast cancer patients in each group randomly frequency matched with distributions of age, and index year at 1 to 1 ratio. Age was set into three categories (18 to 39 years, 40 to 59 years, and more than 60 years). Gender was categorized as male and female. Urbanization was set into four levels (1, highest, 2, 3 and 4, lowest). The Charlson Comorbidity Index (CCI) score was divided into three groups (0, 1, and 2 or higher). Besides, treatment (radiotherapy, chemotherapy, breast cancer surgery) and drugs which patients received within half a year after the new diagnosis of breast cancer were included and followed up to the end of the study.

### 2.3. Study Outcome

All-cause mortality was set as the study outcome, in which date of death was obtained from the major illness certificate. The follow-up time was estimated in person-years, continued until the end of December 2011 or the death of the patient, whichever occurred first.

### 2.4. Statistical Analysis

The CHM user and the non-CHM user were compared in terms of demographic characteristics, CCI score, treatment, and drugs. To exam the categorical variables of the baseline characteristics, chi-square test was used. Otherwise, to exam continuous variables, t test was used. The survival probability between CHM group and non-CHM group were plotted by the Kaplan–Meier method and estimated by the Log-rank test, respectively. To calculate the HRs and 95% confidence intervals (CI) for the breast cancer mortality rate, the Cox proportional hazard model was utilized. SAS statistical software (version 9.4) was used to analyze all the database in the study with a two-tailed significance level of 0.05.

## 3. Results

### 3.1. Baseline Characteristics of Breast Cancer Patients

The baseline characteristics of the CHM user and non-CHM user were displayed in Table 1. In total, 5387 CHM users and 5387 non-CHM users with respective mean ages of 49.35 ± 10.08 and 49.41 ± 10.12 years were recruited in the analysis. In total, 67.94 % of patients were 40–59 years old, and almost all patients (94.45%) had a CCI score of 0. A significantly higher percentages of patients had received previous breast surgery (94.8% vs. 85.74%, *p* < 0.0001), Cyclophosphamide (70.54% vs. 67.27%, *p* = 0.0002), Tamoxifen (69.96% vs. 65.23%, *p* < 0.0001), and Anastrozole (16.3% vs. 12.31%, *p* < 0.0001) in the CHM users. Otherwise, a significantly lower percentages of patients had received Docetaxel (16.87% vs. 21.55%, *p* < 0.0001), Paclitaxel (11.62 vs. 14.52, *p* < 0.0001) and Trastuzumab (6.61% vs. 7.82%, *p* = 0.0155) in the CHM users.

### 3.2. Effect of CHM on Mortality Rate of Breast Cancer Patients

**Figure 1:**
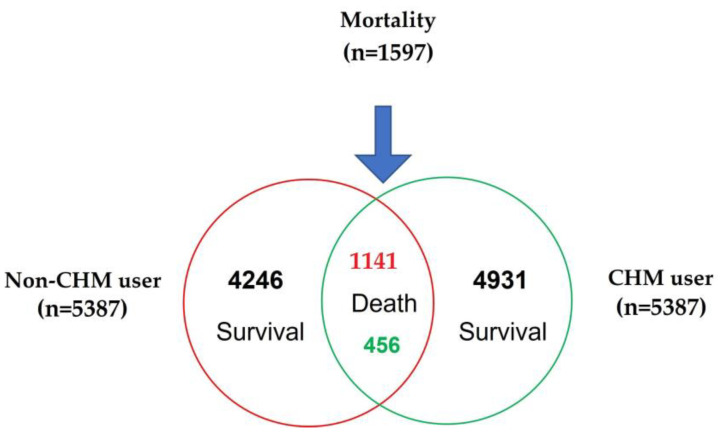
Mortality number in breast cancer patients with and without taking CHM.

At the end of the follow up, 1597 of all enrolled patients had died (456 CHM-users and 1141 non-CHM users) (Figure 1). To elucidate the effect of CHM on breast cancer mortality rate, the cancer mortality rate among CHM user and non-CHM user was stratified according to baseline characteristics of breast cancer patients using the Cox proportional hazard regression model (Table 2). Mortality incidence densities were 13.62 per 1000 person-years and 41.38 per 1000 person-years in the CHM users and non-CHM users, respectively. The mortality rate was significantly (*p* < 0.001) lower in the CHM users (decrease by 59 % after adjusting for age group, urbanization level, CCI score, treatment, and drugs). In age-specific analyses, the CHM users also had a significantly lower mortality rate than non-CHM users in all age groups. In urbanization level or CCI score-specific analyses, the CHM users also had a significantly lower mortality rate than non-CHM users in all groups. Regardless of comorbidities, treatment, and drugs, CHM users had a relatively lower mortality rate. Treatment-stratified analysis revealed that mortality was higher in the treatment group compared to the group without receiving treatment. In sub-analyses of the drug, mortality was higher in the drug group compared to the group not receiving a drug except Toremifene.

**Figure 2:**
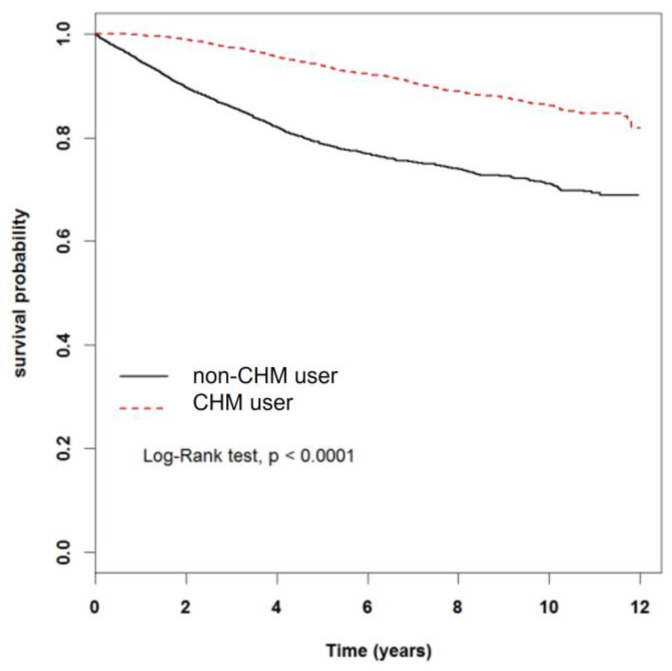
Kaplan–Meier curves for the survival analysis in breast cancer patients with and without taking CHM.

The survival probability between CHM user and non-CHM user among breast cancer patients was plotted in Figure 2. Kaplan–Meier survival analysis with a log rank test revealed that CHM users had significantly higher survival rate compared to non-CHM users (*p* < 0.001).

### 3.3. The Cumulative Days and Annual Average Dose of CHM Use Influence the Breast Cancer Mortality Rate

Next, subgroup analyses were further arranged to clarify the influence of the cumulative days and annual average dose of CHM use among breast cancer patients. The results shows that the risk of cancer mortality rate significantly decreased with the increasing cumulative days of CHM use (HR = 0.44, 0.41 and 0.31 in 30–90 days, 90–180 days, and >180 days, respectively, *p* for trend <0.001) (Table 3). In total, 25, 50, and 75 percentiles of the annual average CHM dose were 35.1, 67.2, and 147 (g), respectively. The risk of cancer mortality rate significantly decreased with the increasing annual average CHM dose (HR = 0.50, 0.43, 0.39, and 0.30 at <35.1 g, 35.1–67.2 g, 67.2–147 g, and >147 g, respectively, *p* for trend <0.001) (Table 4). Relative to non-CHM users, the cancer mortality rate in CHM users was the lowest during follow-up periods <2 years and then increased with an increasing follow-up period. However, the mortality rate did not significantly differ between CHM users and non-CHM users after a follow-up of more than 5 years (Table 5). Taken together, the protective effect of CHM was parallel to the cumulative days of CHM use and the annual average CHM dose, which is most dominant during the initial usage.

### 3.4. The Impact of SHXXT and Its Constituents on the Breast Cancer Mortality Rate

At last, to clarify the effect of SHXXT and its constituents among breast cancer patients, stratified analyses were performed further. The results reveals that the mortality rate was lower in patients who used SHXXT compound compared to patients who only used one of its constituents (HR = 0.42, 0.40, 0.39 and 0.32 for *Rhizoma Rhei*, *Radix Scutellaria*, *Rhizoma Coptidis*, and SHXXT, respectively) (Table 6). Above all, SHXXT compound elicited the more significant impact on the deceasing cancer mortality rate compared to the use of one individual constituents.

## 4. Discussion

This retrospective, large-scale, population-based cohort study is not only the first Taiwanese study to investigate the cancer mortality rate among breast cancer patients who received SHXXT or its constituents but also the first study in the Asian population. The use of SHXXT or its constituents reduced the breast cancer mortality rate by 59%. Notably, the protective effect of SHXXT and its constituents on breast cancer increased with the cumulative days and annual average dose. Moreover, the mortality rate was lower in breast cancer patients who used the SHXXT compound than those who only used one of its constituents.

Series studies have reported that extracts of SHXXT constituents have anti-cancer effects. According to the gene set enrichment analysis by Cheng et al., SHXXT can exert anti-proliferative effects on breast cancer HepG2 cells through regulating p53 signaling [26]. The decoction SHXXT typically contains *Rhizoma Rhei*, *Radix Scutellaria*, and *Rhizoma Coptidis* at ratios of 2:1:1 or 1:1:1. Notably, the anti-proliferative effects of SHXXT are mainly conferred by *Rhizoma Coptidis*. The major component of *Rhizoma Coptidis* is berberine; it can induce human breast cancer cell apoptosis by upregulating TNF-α and interferon-β in a time- or dose-dependent manner [29,30]. Chou and their colleagues reported that berberine induces cytotoxicity in breast cancer cells through reactive oxygen species, dysregulation of redox regulation, centrosomal structure, electron transport, cell signaling, protein folding, proteolysis, and protein trafficking [31]. Berberine decreases cell viability by inhibiting the Ras/MAPK and PI3K pathways, by suppressing activation of growth factor receptors (Her2/neu, EGF receptor, and VEGF receptor), by increasing apoptosis-related molecules (Smac/DIABLO, caspases, and Bax), tumor suppressor genes (p53, Clip/p21, Klip/p27, and Rb) [32], and over-expression of HDAC1 [33], prohibitin [34], serine/threonine protein phosphatase 2A [35,36], and by blockade of Bcl-2 expression. In addition, berberine can increase the amount of E-cadherin and suppress activation of the Wnt/ β-catenin pathway to modulate tumor cell migration and metastasis [37]. Apart from berberine, coptisine is an isoquinoline alkaloid extract of *Rhizoma Coptidis*, from which can emerge the anti-metastasis effect. Through down-regulating MMP-9 and increasing TIMP-1, coptisine can suppress adhesion, migration, and invasion of MDA-MB-231 breast cancer cells [38]. Hence, *Rhizoma Coptidis* inhibits the breast cancer cell proliferation and metastasis based on these two important components.

In addition to *Rhizoma Coptidis*, *Radix Scutellaria* is another constituent of SHXXT, which also has an anti-cancer effect due to its containing Oroxylin A and numerous flavonoids such as wogonin and baicalin [39]. Being a major component of *Radix Scutellaria*, Oroxylin A inhibits the binding activity of hexokinase-II (HK-II) with mitochondria in a SIRT3-dependent manner to suppress glycolysis and induce mitochondrial cytotoxicity in human breast cancer cell lines [40]. Another component of *Radix Scutellaria*, wogonin, inhibits breast cancer cells invasion by downregulating ERK1/2 and PKC-δ [41]. Through decreasing both endogenous and PMA-induced MMP-9 expression, wogonin could inhibit tumor invasion and metastasis [42]. Wogonin also could induce cell differentiation, impair cell apoptosis, and activate both antioxidant and anti-angiogenesis activity against breast cancer formation [43,44,45,46].

Moreover, the other constituent of SHXXT, *Rhizoma Rhei*, exerts a high estrogenic potency, of which chrysophanol 1-O-β-D-glucopyranoside, aloe emodin, and rhapontigenin are the main isolated compounds. Most breast cancer cells are estrogen receptor- (ER)/progesterone receptor (PR)-positive, with human epidermal growth factor receptor 2 (HER2)-negative (69%), and almost half of postmenopausal patients have ER-positive breast cancer cells [47,48]. Since estrogen can stimulate ER-positive breast cancer cell to proliferate, a selective ER modulator that blocks the binding of estrogen to ER is effective to treat ER-positive breast cancer [49,50]. Through regulating the expression of ER, *Rhizoma Rhei* can modulate the proliferation of breast cancer MCF-7 cell in a concentration-dependent manner [51]. Chrysophanol 1-O-β-D-glucopyranoside mediates mitochondria-dependent apoptosis, whereas aloe emodin and rhapontigenin activate the caspase-8 pathway to induce mitochondria-independent apoptosis. Therefore, *Rhizoma Rhei* components could help hormone replacement therapy and chemoprevention against breast cancer owing to their potent estrogenic and inhibitory activities [52].

Since three constituents of SHXXT all have an important anti-cancer effect, the cancer mortality rate influence of the SHXXT component is supposed to be superior to that of a single constituent. Around the world, the major causes of morbidity and mortality are tumor invasion and metastasis. To halt the breast cancer mortality, the main objective is to inhibit cancer invasion and metastasis. Since extracts of three SHXXT constituents not only have anti-proliferative and anti-angiogenesis effects but also exert anti-invasive and anti-metastatic impacts on breast cancer, the multi-component nature of medicinal herbs makes them particularly suitable to treat complex disease through synergistic activity [53]. Therefore, SHXXT is more effective than its individual components in terms of reducing mortality in breast cancer patients.

The strength of the study is that the study traced a large sample size of patients for a long period by using NHIRD. NHIRD is good for assessing survival rates, herb–drug interactions, and the cost-effectiveness of drug treatments. In addition, the study analyzed the specific ascertainment of numerous outcome events, the identification of an exposure–response relationship, and the comparison of different average annual doses. Besides, the data contained in an administrative database rather than data collected in a hospital-based study helped to avoid selection bias.

However, several limitations are retained in the study. First, this retrospective observational study could only analyze the data contained in NHIRD. Since the de-identified data are released for public research and the NHIRD is a claims-based database, no additional clinical information is available, e.g., histology subtype, gene change, and cancer staging [54,55,56]. Second, some possible confounders such as lifestyle practices, physical activity, dietary intake, and genetic factors are unavailable. Besides, the exposure–response relationship is only expressed in terms of cumulative days of CHM use; the actual adherence could not be ascertained. In addition, patients directly purchasing CHM or herbs from herbal pharmacies or health food stores cannot be identified in the NHI program. Only CHM prescribed by licensed doctors can be reimbursed; hence, the frequency of the CHM use might have been under-estimated. However, a large portion of patients would likely prefer to purchase CHM in the NHI system because of the comprehensive coverage and lower cost of CHM prescriptions. Finally, randomized controlled trials should be arranged in the future due to the more powerful statistical significance.

## 5. Conclusions

Our study suggests that exposure to CHM and SHXXT, especially a high annual average dose, or high cumulative use days, is associated with reduced mortality in breast cancer patients. Patients receiving SHXXT for more than 30 days as a treatment apparently seem to have a better overall survival outcome than do patients who did not receive it more than 30 days. However, mortality increased with a follow-up duration at all timepoints except >5 years. Hence, SHXXT and its individual compounds can be considered promising therapeutic weapons against breast cancer. Although the detailed mechanisms are as yet elusive, SHXXT is generally considered the stronger anti-inflammatory medicine, or the so-called “clear the heat”, in the Chinese Medicine system. Therefore, we are now performing ongoing clinical trials at Kaohsiung medical university hospital which were approved by the Institutional Review Board of Kaohsiung Medical University Hospital to investigate the effects of SHXXT intervention in patients who suffer from breast cancer.

## Figures and Tables

**Table 1:** Baseline characteristics of breast cancer patients.

Characteristics	Non-CHM User (n = 5387)	CHM User (n = 5387)	*p*-Value
**Age, mean ± SD (years)**	49.41 (10.12)	49.35 (10.08)	0.7591
**Age group, n (%)**			0.99
18–39	911 (16.91)	911 (16.91)	
40–59	3660 (67.94)	3660 (67.94)	
≥60	816 (15.15)	816 (15.15)	
**Urbanization level, n (%)**			<0.0001
1	2002 (37.16)	1790 (33.23)	
2	1724 (32)	1784 (33.12)	
3	725 (13.46)	879 (16.32)	
4	936 (17.38)	934 (17.34)	
**CCI score, n (%)**			0.0006
0	5044 (96.63)	5088 (94.45)	
1	201 (3.73)	214 (3.97)	
More than 2	142 (2.64)	85 (1.58)	
**Treatment, n (%)**			
Breast cancer surgery	4619 (85.74)	5107 (94.8)	<.0001
Radiotherapy	2666 (49.49)	2770 (51.42)	0.0451
Chemotherapy	3936(73.06)	3958 (73.47)	0.632
**Drugs, n (%)**			
Epirubicin	2179 (40.45)	2207 (40.97)	0.583
Fluorouracil	3082 (57.21)	3126 (58.03)	0.391
Cyclophosphamide	3624 (67.27)	3800 (70.54)	0.0002
Docetaxel	1161 (21.55)	909 (16.87)	<0.0001
Paclitaxel	782 (14.52)	626 (11.62)	<0.0001
Goserelin acetate	67 (1.24)	61 (1.13)	0.5937
Tamoxifen	3514 (65.23)	3769 (69.96)	<0.0001
Anastrozole	663 (12.31)	878 (16.3)	<0.0001
Letrozole	771 (14.31)	824 (15.3)	0.1505
Exemestane	413 (7.67)	405 (7.52)	0.7711
Toremifene	52 (0.97)	64 (1.19)	0.2626
Trastuzumab	421 (7.82)	356 (6.61)	0.0155

Abbreviations: CHM, Chinese herbal medicine; CCI, Charlson Comorbidity Index.

**Table 2:** Breast cancer mortality rate among CHM user and non-CHM user stratified by age group, urbanization level, CCI score, treatment, and drugs.

Characteristics	Non-CHM User			CHM User			Crude HR	Adjusted HR ^‡^
	Event	Person Years	IR ^†^	Event	Person Years	IR ^†^		
**Total**	1141	27,576	41.38	456	33,488	13.62	0.33 (0.3–0.37) ***	0.41 (0.37–0.46) ***
**Age group**								
18–39	171	4879	34.92	75	6083	12.33	0.35 (0.27–0.46) ***	0.4 (0.3–0.54) ***
40–59	741	18,929	39.15	288	22,641	12.72	0.33 (0.29–0.38) ***	0.41 (0.35–0.47) ***
≥60	229	3750	61.07	93	4765	19.52	0.33 (0.26–0.42) ***	0.35 (0.27–0.45) ***
Urbanization level								
1	348	10,701	32.52	138	11,097	12.44	0.39 (0.32–0.47) ***	0.49 (0.4–0.6) ***
2	368	8786	41.88	158	11,069	14.27	0.34 (0.28–0.41) ***	0.38 (0.31–0.46) ***
3	185	3512	52.68	71	5438	13.06	0.25 (0.19–0.33) ***	0.33 (0.25–0.43) ***
4	240	4577	52.43	89	5884	15.13	0.3 (0.23–0.38) ***	0.39 (0.3–0.5) ***
**CCI score**								
0	1012	26,100	38.77	416	31,838	13.07	0.34 (0.3–0.38) ***	0.41 (0.37–0.46) ***
1	70	937	74.73	21	1240	16.93	0.23 (0.14–0.37) ***	0.29 (0.17–0.49) ***
More than 2	59	539	109.42	19	410	46.30	0.43 (0.26–0.73) **	0.42 (0.24–0.73) **
**Treatment**								
Breast cancer surgery								
No	367	2704	135.74	48	1903	25.22	0.23 (0.17–0.32) ***	0.25 (0.18–0.34) ***
Yes	774	24,872	31.12	408	31,585	12.92	0.41 (0.37–0.46) ***	0.43 (0.38–0.49) ***
Radiotherapy								
No	426	14,848	28.69	116	17,493	6.63	0.24 (0.2–0.3) ***	0.29 (0.24–0.36) ***
Yes	715	12,728	56.18	340	15,996	21.26	0.37 (0.33–0.43) ***	0.46 (0.41–0.53) ***
Chemotherapy								
No	177	7689	23.02	37	9584	3.86	0.18 (0.13–0.26) ***	0.2 (0.14–0.28) ***
Yes	964	19,887	48.87	419	23,904	17.53	0.36 (0.32–0.41) ***	0.45 (0.4–0.5) ***
**Drugs**								
Epirubicin								
No	567	17.185	32.99	182	20.655	8.81	0.27 (0.23–0.32) ***	0.35 (0.3–0.42) ***
Yes	574	10.391	55.24	274	12.834	21.35	0.38 (0.33–0.44) ***	0.44 (0.38–0.51) ***
Fluorouracil								
No	383	11.574	33.09	124	13.796	8.99	0.28 (0.23–0.34) ***	0.35 (0.28–0.43) ***
Yes	758	16.002	47.37	332	19.692	16.86	0.36 (0.31–0.4) ***	0.42 (0.37–0.48) ***
Cyclophosphamide								
No	332	8825	37.62	83	10.396	7.98	0.23 (0.18–0.29) ***	0.29 (0.22–0.37) ***
Yes	809	18.751	43.14	373	22.093	16.15	0.37 (0.33–0.42) ***	0.44 (0.38–0.49) ***
Docetaxel								
No	610	23.279	26.20	196	29.218	6.71	0.26 (0.22–0.31) ***	0.31 (0.26–0.36) ***
Yes	531	4297	123.57	260	4270	60.89	0.46 (0.39–0.53) ***	0.52 (0.45–0.61) ***
Paclitaxel								
No	722	24.227	29.80	228	30.102	7.57	0.26 (0.23–0.3) ***	0.31 (0.27–0.36) ***
Yes	419	3349	125.12	228	3387	67.32	0.5 (0.43–0.59) ***	0.55 (0.47–0.65) ***
Goserelin acetate								
No	1133	27.222	40.89	440	33.156	13.27	0.33 (0.29–0.37) ***	0.4 (0.36–0.45) ***
Yes	28	354	79.19	16	332	48.14	0.59 (0.32–1.09)	0.49 (0.22–1.07)
Tamoxifen								
No	526	8274	63.57	147	9402	15.64	0.26 (0.22–0.31) ***	0.34 (0.28–0.41) ***
Yes	615	19.302	31.86	309	24.087	12.83	0.4 (0.35–0.46) ***	0.45 (0.39–0.52) ***
Anastrozole								
No	895	23.944	37.38	299	27.694	10.80	0.3 (0.26–0.34) ***	0.37 (0.32–0.42) ***
Yes	246	3632	67.74	157	5795	27.09	0.39 (0.32–0.47) ***	0.5 (0.4–0.61) ***
Letrozole								
No	898	23.522	38.18	295	28.283	10.43	0.28 (0.25–0.32) ***	0.35 (0.3–0.39) ***
Yes	243	4054	59.94	161	5206	30.93	0.49 (0.4–0.6) ***	0.6 (0.49–0.74) ***
Exemestane								
No	962	25.296	38.03	337	30.880	10.91	0.29 (0.26–0.33) ***	0.37 (0.32–0.42) ***
Yes	179	2280	78.49	119	2609	45.62	0.55 (0.44–0.7) ***	0.58 (0.46–0.74) ***
Toremifene								
No	1123	27.243	41.22	447	33.027	13.53	0.33 (0.3–0.37) ***	0.41 (0.36–0.45) ***
Yes	18	333	54.05	9	461	19.50	0.35 (0.16–0.78) **	0.11 (0.02–0.48) **
Trastuzumab								
No	934	26.041	35.87	347	31.897	10.88	0.31 (0.27–0.35) ***	0.39 (0.34–0.44) ***
Yes	207	1535	134.90	109	1591	68.50	0.46 (0.37–0.58) ***	0.48 (0.38–0.61) ***

Abbreviations: CHM, Chinese herbal medicine; CCI, Charlson Comorbidity Index; HR, hazard ratio; CI, confidence interval. † IR, incidence rates, per 1000 person-years. ‡: represented adjusted hazard ratio: mutually adjusted for CHM use, age group, urbanization level, CCI score, treatment, and drugs by Cox proportional hazard regression. ** *p* < 0.01, *** *p* < 0.001.

**Table 3:** The risk of mortality rate stratified by the cumulative days of CHM use among breast cancer patients.

Characteristics	N	Mortality	HR (95% CI)	
		No. of Event	Crude	Adjusted ^†^
**Non-CHM users**	5387	1141	1 (reference)	1 (reference)
**CHM users**				
**30–90 days**	3209	288	0.36 (0.31–0.40) ***	0.44 (0.38–0.50) ***
**90–180 days**	1233	106	0.33 (0.27–0.41) ***	0.41 (0.33–0.50) ***
**>180 days**	945	62	0.26 (0.20–0.33) ***	0.31 (0.24–0.40) ***
***p* for trend**			**<0.0001**	**<0.0001**

Abbreviations: CHM, Chinese herbal medicine; HR, hazard ratio; CI, confidence interval. Crude HR represented relative hazard ratio. † Adjusted HR represented adjusted hazard ratio: mutually adjusted for age group, urbanization level, CCI score, treatment, and drugs by Cox proportional hazard regression. *** *p* < 0.001.

**Table 4:** The risk of mortality rate stratified by the annual average CHM dose among breast cancer patients.

Annual Average CHM Dose (g)	N	Mortality	HR (95% CI)	
		No. of Event	Crude	Adjusted ^†^
**Non-CHM users**	5387	1141	1 (reference)	1 (reference)
**CHM users**				
**<35.1 (g)/year**	1346	140	0.42 (0.35–0.50) ***	0.50 (0.42–0.60) ***
**35.1–67.2 (g)/year**	1346	123	0.36 (0.30–0.44) ***	0.43 (0.35–0.51) ***
**67.2–147 (g)/year**	1340	113	0.33 (0.27–0.40) ***	0.39 (0.32–0.48) ***
**>147 (g)/year**	1355	80	0.23 (0.18–0.29) ***	0.30 (0.24–0.38) ***
***p* for trend**			<0.0001	<0.0001

Abbreviations: CHM, Chinese herbal medicine; HR, hazard ratio; CI, confidence interval. Crude HR represented relative hazard ratio. † Adjusted HR represented adjusted hazard ratio: mutually adjusted for age group, urbanization level, CCI score, treatment, and drugs by Cox proportional hazard regression. *** *p* < 0.001.

**Table 5:** The effects of the cumulative days of CHM use on the mortality rate among breast cancer patients stratified by follow-up period.

Characteristics	Mortality	HR (95% CI)	
	No. of Event	Crude	Adjusted ^†^
**Follow-up period:** **≤** **2 years**			
**Non-CHM users**	440	1 (reference)	1 (reference)
**CHM users**			
** 30–90 days**	136	0.43 (0.35–0.52) ***	0.51 (0.42–0.62) ***
** 90–180 days**	52	0.42 (0.31–0.56) ***	0.45 (0.34–0.61) ***
** >180 days**	24	0.25 (0.17–0.38) ***	0.29 (0.19–0.44) ***
**Follow-up period: 2–5 years**			
**Non-CHM users**	137	1 (reference)	1 (reference)
**CHM users**			
** 30–90 days**	91	0.82 (0.63–1.07)	0.88 (0.68–1.15)
** 90–180 days**	37	0.83 (0.58–1.20)	0.82 (0.57–1.19)
** >180 days**	28	0.87 (0.58–1.31)	0.82 (0.55–1.24)
**Follow-up period: >5 years**			
**Non-CHM users**	25	1 (reference)	1 (reference)
**CHM users**			
** 30–90 days**	16	0.70 (0.37–1.31)	0.60 (0.31–1.51)
** 90–180 days**	6	0.69 (0.28–1.67)	0.58 (0.22–1.57)
** >180 days**	8	1.28 (0.58–2.83)	0.75 (0.32–1.78)

Abbreviations: CHM, Chinese herbal medicine; HR, hazard ratio; CI, confidence interval. Crude HR represented relative hazard ratio. † Adjusted HR represented adjusted hazard ratio: mutually adjusted for age group, urbanization level, CCI score, treatment, and drugs by Cox proportional hazard regression. *** *p* < 0.001.

**Table 6:** The effects of CHM formulation on mortality rate among breast cancer patients.

CHM Prescription	Mortality		HR (95% CI)	
	N	No. of Event	Crude	Adjusted ^†^
**Non-CHM user**	5387	1141	1 (reference)	1 (reference)
**Single constituent**				
***Rhizoma Rhei***	3049	278	0.36 (0.31–0.41) ***	0.42 (0.37–0.48) ***
***Radix Scutellaria***	3958	327	0.32 (0.28–0.36) ***	0.40 (0.36–0.46) ***
***Rhizoma Coptidis***	2644	215	0.31 (0.27–0.36) ***	0.39 (0.34–0.45) ***
**Compounds**				
**SHXXT**	489	33	0.25 (0.18–0.36) ***	0.32 (0.22–0.45) ***

Abbreviations: CHM, Chinese herbal medicine; HR, hazard ratio; CI, confidence interval. Crude HR represented relative hazard ratio. † Adjusted HR represented adjusted hazard ratio: mutually adjusted for age group, urbanization level, CCI score, treatment, and drugs by Cox proportional hazard regression. *** *p* < 0.001.

## Data Availability

All data generated or analyzed during this study are included in this published article.

## References

[1] Youlden D.R., Cramb S.M., Dunn N.A., Muller J.M., Pyke C.M., Baade P.D. (2012). The descriptive epidemiology of female breast cancer: An international comparison of screening, incidence, survival and mortality. Cancer Epidemiol..

[2] Kamangar F., Dores G.M., Anderson W.F. (2006). Patterns of cancer incidence, mortality, and prevalence across five continents: Defining priorities to reduce cancer disparities in different geographic regions of the world. J. Clin. Oncol..

[3] Banas T., Juszczyk G., Pitynski K., Nieweglowska D., Ludwin A., Czerw A. (2016). Incidence and mortality rates in breast, corpus uteri, and ovarian cancers in poland (1980–2013): An analysis of population-based data in relation to socioeconomic changes. OncoTargets Ther..

[4] Liu F.C., Lin H.T., Kuo C.F., See L.C., Chiou M.J., Yu H.P. (2017). Epidemiology and survival outcome of breast cancer in a nationwide study. Oncotarget.

[5] Chuang S.C., Wu G.J., Lu Y.S., Lin C.H., Hsiung C.A. (2015). Associations between medical conditions and breast cancer risk in asians: A nationwide population-based study in taiwan. PLoS ONE.

[6] Figueroa-Magalhaes M.C., Jelovac D., Connolly R., Wolff A.C. (2014). Treatment of her2-positive breast cancer. Breast.

[7] Hu C., Zhang H., Wu W., Yu W., Li Y., Bai J., Luo B., Li S. (2016). Acupuncture for pain management in cancer: A systematic review and meta-analysis. Evid. Based Complement. Altern. Med. ECAM.

[8] Chung V.C., Wu X., Lu P., Hui E.P., Zhang Y., Zhang A.L., Lau A.Y., Zhao J., Fan M., Ziea E.T. (2016). Chinese herbal medicine for symptom management in cancer palliative care: Systematic review and meta-analysis. Medicine.

[9] Pu C.Y., Lan V.M., Lan C.F., Lang H.C. (2008). The determinants of traditional chinese medicine and acupuncture utilization for cancer patients with simultaneous conventional treatment. Eur. J. Cancer Care.

[10] Kuo Y.T., Chang T.T., Muo C.H., Wu M.Y., Sun M.F., Yeh C.C., Yen H.R. (2018). Use of complementary traditional chinese medicines by adult cancer patients in taiwan: A nationwide population-based study. Integr. Cancer Ther..

[11] Tsai Y.T., Lai J.N., Lo P.C., Chen C.N., Lin J.G. (2017). Prescription of chinese herbal products is associated with a decreased risk of invasive breast cancer. Medicine.

[12] Lin Y.H., Chiu J.H. (2011). Use of chinese medicine by women with breast cancer: A nationwide cross-sectional study in taiwan. Complement. Ther. Med..

[13] Coussens L.M., Werb Z. (2002). Inflammation and cancer. Nature.

[14] Wu J., Hu Y., Xiang L., Li S., Yuan Y., Chen X., Zhang Y., Huang W., Meng X., Wang P. (2016). San-huang-xie-xin-tang constituents exert drug-drug interaction of mutual reinforcement at both pharmacodynamics and pharmacokinetic level: A review. Front. Pharmacol..

[15] Lee J.C., Tseng C.K., Wu S.F., Chang F.R., Chiu C.C., Wu Y.C. (2011). San-huang-xie-xin-tang extract suppresses hepatitis c virus replication and virus-induced cyclooxygenase-2 expression. J. Viral Hepat..

[16] Chen H.C., Hsieh M.T. (1986). Two-year experience with “san-huang-hsieh-hsin-tang” in essential hypertension. Am. J. Chin. Med..

[17] Chen H.C., Hsieh M.T., Tsai H.Y., Chang H.H., Wang T.F., Shibuya T. (1984). Studies on the “san-huang-hsieh-hsin-tang” in the treatment of essential hypertension. Taiwan Yi Xue Hui Za Zhi.

[18] Tsai H.H., Chen I.J., Lo Y.C. (2008). Effects of san-huang-xie-xin-tang on u46619-induced increase in pulmonary arterial blood pressure. J. Ethnopharmacol..

[19] Lo Y.C., Tsai P.L., Huang Y.B., Shen K.P., Tsai Y.H., Wu Y.C., Lai Y.H., Chen I.J. (2005). San-huang-xie-xin-tang reduces lipopolysaccharides-induced hypotension and inflammatory mediators. J. Ethnopharmacol..

[20] Shih Y.T., Chen I.J., Wu Y.C., Lo Y.C. (2011). San-huang-xie-xin-tang protects against activated microglia- and 6-ohda-induced toxicity in neuronal sh-sy5y cells. Evid. Based Complement. Altern. Med..

[21] Hwang M.W., Ahn T.S., Hong N.R., Jeong H.S., Jung M.H., Ha K.T., Kim B.J. (2015). Effects of traditional chinese herbal medicine san-huang-xie-xin-tang on gastrointestinal motility in mice. World J. Gastroenterol..

[22] Shih Y.T., Wu D.C., Liu C.M., Yang Y.C., Chen I.J., Lo Y.C. (2007). San-huang-xie-xin-tang inhibits helicobacter pylori-induced inflammation in human gastric epithelial ags cells. J. Ethnopharmacol..

[23] Lo Y.C., Lin Y.L., Yu K.L., Lai Y.H., Wu Y.C., Ann L.M., Chen I.J. (2005). San-huang-xie-xin-tang attenuates inflammatory responses in lipopolysaccharide-exposed rat lungs. J. Ethnopharmacol..

[24] Liou S.F., Ke H.J., Hsu J.H., Liang J.C., Lin H.H., Chen I.J., Yeh J.L. (2011). San-huang-xie-xin-tang prevents rat hearts from ischemia/reperfusion-induced apoptosis through enos and mapk pathways. Evid. Based Complement. Altern. Med..

[25] Li C.Y., Hou Y.C., Lee Chao P.D., Shia C.S., Hsu I.C., Fang S.H. (2010). Potential ex vivo immunomodulatory effects of san-huang-xie-xin-tang and its component herbs on mice and humans. J. Ethnopharmacol..

[26] Cheng W.Y., Wu S.L., Hsiang C.Y., Li C.C., Lai T.Y., Lo H.Y., Shen W.S., Lee C.H., Chen J.C., Wu H.C. (2008). Relationship between san-huang-xie-xin-tang and its herbal components on the gene expression profiles in hepg2 cells. Am. J. Chin. Med..

[27] Shia C.S., Hou Y.C., Juang S.H., Tsai S.Y., Hsieh P.H., Ho L.C., Chao P.D. (2011). Metabolism and pharmacokinetics of san-huang-xie-xin-tang, a polyphenol-rich chinese medicine formula, in rats and ex-vivo antioxidant activity. Evid. Based Complement. Altern. Med. ECAM.

[28] Lo Y.C., Shih Y.T., Tseng Y.T., Hsu H.T. (2012). Neuroprotective effects of san-huang-xie-xin-tang in the mpp(+)/mptp models of parkinson’s disease in vitro and in vivo. Evid. Based Complement. Altern. Med. ECAM.

[29] Li X.K., Motwani M., Tong W., Bornmann W., Schwartz G.K. (2000). Huanglian, a chinese herbal extract, inhibits cell growth by suppressing the expression of cyclin b1 and inhibiting cdc2 kinase activity in human cancer cells. Mol. Pharmacol..

[30] Kang J.X., Liu J., Wang J., He C., Li F.P. (2005). The extract of huanglian, a medicinal herb, induces cell growth arrest and apoptosis by upregulation of interferon-beta and tnf-alpha in human breast cancer cells. Carcinogenesis.

[31] Chou H.C., Lu Y.C., Cheng C.S., Chen Y.W., Lyu P.C., Lin C.W., Timms J.F., Chan H.L. (2012). Proteomic and redox-proteomic analysis of berberine-induced cytotoxicity in breast cancer cells. J. Proteom..

[32] Gu W., Luo J., Brooks C.L., Nikolaev A.Y., Li M. (2004). Dynamics of the p53 acetylation pathway. Novartis Found. Symp..

[33] Cai R.L., Yan-Neale Y., Cueto M.A., Xu H., Cohen D. (2000). Hdac1, a histone deacetylase, forms a complex with hus1 and rad9, two g2/m checkpoint rad proteins. J. Biol. Chem..

[34] McClung J.K., Jupe E.R., Liu X.T., Dell’Orco R.T. (1995). Prohibitin: Potential role in senescence, development, and tumor suppression. Exp. Gerontol..

[35] Klumpp S., Krieglstein J. (2002). Serine/threonine protein phosphatases in apoptosis. Curr. Opin. Pharmacol..

[36] Garcia A., Cayla X., Guergnon J., Dessauge F., Hospital V., Rebollo M.P., Fleischer A., Rebollo A. (2003). Serine/threonine protein phosphatases pp1 and pp2a are key players in apoptosis. Biochimie.

[37] Qi H.W., Xin L.Y., Xu X., Ji X.X., Fan L.H. (2014). Epithelial-to-mesenchymal transition markers to predict response of berberine in suppressing lung cancer invasion and metastasis. J. Transl. Med..

[38] Li J., Qiu D.M., Chen S.H., Cao S.P., Xia X.L. (2014). Suppression of human breast cancer cell metastasis by coptisine in vitro. Asian Pac. J. Cancer Prev. APJCP.

[39] Yu C.P., Hsieh Y.C., Shia C.S., Hsu P.W., Chen J.Y., Hou Y.C., Hsieh Y.W. (2016). Increased systemic exposure of methotrexate by a polyphenol-rich herb via modulation on efflux transporters multidrug resistance-associated protein 2 and breast cancer resistance protein. J. Pharm. Sci..

[40] Wei L., Zhou Y., Dai Q., Qiao C., Zhao L., Hui H., Lu N., Guo Q.L. (2013). Oroxylin a induces dissociation of hexokinase ii from the mitochondria and inhibits glycolysis by sirt3-mediated deacetylation of cyclophilin d in breast carcinoma. Cell Death Dis..

[41] Zhang J., Anastasiadis P.Z., Liu Y., Thompson E.A., Fields A.P. (2004). Protein kinase c (pkc) betaii induces cell invasion through a ras/mek-, pkc iota/rac 1-dependent signaling pathway. J. Biol. Chem..

[42] Chen P., Lu N., Ling Y., Chen Y., Hui H., Lu Z., Song X., Li Z., You Q., Guo Q. (2011). Inhibitory effects of wogonin on the invasion of human breast carcinoma cells by downregulating the expression and activity of matrix metalloproteinase-9. Toxicology.

[43] Lim B.O. (2003). Effects of wogonin, wogonoside, and 3,5,7,2’,6’-pentahydroxyflavone on chemical mediator production in peritoneal exduate cells and immunoglobulin e of rat mesenteric lymph node lymphocytes. J. Ethnopharmacol..

[44] Wang W., Guo Q., You Q., Zhang K., Yang Y., Yu J., Liu W., Zhao L., Gu H., Hu Y. (2006). Involvement of bax/bcl-2 in wogonin-induced apoptosis of human hepatoma cell line smmc-7721. Anti Cancer Drugs.

[45] Tai M.C., Tsang S.Y., Chang L.Y., Xue H. (2005). Therapeutic potential of wogonin: A naturally occurring flavonoid. CNS Drug Rev..

[46] Lu N., Gao Y., Ling Y., Chen Y., Yang Y., Gu H.Y., Qi Q., Liu W., Wang X.T., You Q.D. (2008). Wogonin suppresses tumor growth in vivo and vegf-induced angiogenesis through inhibiting tyrosine phosphorylation of vegfr2. Life Sci..

[47] Zingue S., Nde C.B.M., Michel T., Ndinteh D.T., Tchatchou J., Adamou M., Fernandez X., Fohouo F.T., Clyne C., Njamen D. (2017). Ethanol-extracted cameroonian propolis exerts estrogenic effects and alleviates hot flushes in ovariectomized wistar rats. BMC Complement. Altern. Med..

[48] Oumarou M.R., Zingue S., Bakam B.Y., Ateba S.B., Foyet S.H., Mbakop F.T.T., Njamen D. (2017). Lannea acida a. Rich. (anacardiaceae) ethanol extract exhibits estrogenic effects and prevents bone loss in an ovariectomized rat model of osteoporosis. Evid. Based Complement. Altern. Med. ECAM.

[49] Leung E., Kim J.E., Askarian-Amiri M., Finlay G.J., Baguley B.C. (2014). Evidence for the existence of triple-negative variants in the mcf-7 breast cancer cell population. BioMed Res. Int..

[50] Tung N., Wang Y., Collins L.C., Kaplan J., Li H., Gelman R., Comander A.H., Gallagher B., Fetten K., Krag K. (2010). Estrogen receptor positive breast cancers in brca1 mutation carriers: Clinical risk factors and pathologic features. Breast Cancer Res. BCR.

[51] Kim I.G., Kang S.C., Kim K.C., Choung E.S., Zee O.P. (2008). Screening of estrogenic and antiestrogenic activities from medicinal plants. Environ. Toxicol. Pharmacol..

[52] Lee D., Park S., Choi S., Kim S.H., Kang K.S. (2018). In vitro estrogenic and breast cancer inhibitory activities of chemical constituents isolated from rheum undulatum L. Molecules.

[53] Yang Y., Zhang Z., Li S., Ye X., Li X., He K. (2014). Synergy effects of herb extracts: Pharmacokinetics and pharmacodynamic basis. Fitoterapia.

[54] Lee Y.W., Chen T.L., Shih Y.R., Tsai C.L., Chang C.C., Liang H.H., Tseng S.H., Chien S.C., Wang C.C. (2014). Adjunctive traditional chinese medicine therapy improves survival in patients with advanced breast cancer: A population-based study. Cancer.

[55] Lin H.C., Lin C.L., Huang W.Y., Shangkuan W.C., Kang B.H., Chu Y.H., Lee J.C., Fan H.C., Kao C.H. (2015). The use of adjunctive traditional chinese medicine therapy and survival outcome in patients with head and neck cancer: A nationwide population-based cohort study. QJM.

[56] Kuo Y.T., Liao H.H., Chiang J.H., Wu M.Y., Chen B.C., Chang C.M., Yeh M.H., Chang T.T., Sun M.F., Yeh C.C. (2018). Complementary chinese herbal medicine therapy improves survival of patients with pancreatic cancer in taiwan: A nationwide population-based cohort study. Integr. Cancer.

